# Formyl Met-Leu-Phe-Stimulated FPR1 Phosphorylation in Plate-Adherent Human Neutrophils: Enhanced Proteolysis but Lack of Inhibition by Platelet-Activating Factor

**DOI:** 10.1155/2018/3178970

**Published:** 2018-01-24

**Authors:** Algirdas J. Jesaitis, Jeannie Gripentrog, Jovanka M. Voyich

**Affiliations:** Department of Microbiology and Immunology, Montana State University, Bozeman, MT 59717-3520, USA

## Abstract

N-formyl-Met-Leu-Phe (fMLF) is a model PAMP/DAMP driving human PMN to sites of injury/infection utilizing the GPCR, FPR1. We examined a microtiter plate format for measurement of FPR1 phosphorylation in adherent PMN at high densities and found that a new phosphosensitive FPR1 fragment, 25K-FPR1, accumulates in SDS-PAGE extracts. 25K-FPR1 is fully inhibited by diisopropylfluorophosphate PMN pretreatment but is not physiologic, as its formation failed to be significantly perturbed by ATP depletion, time and temperature of adherence, or adherence mechanism. 25K-FPR1 was minimized by extracting fMLF-exposed PMN in lithium dodecylsulfate at 4°C prior to reduction/alkylation. After exposure of adherent PMN to a 5 log range of PAF before or after fMLF, unlike in suspension PMN, no inhibition of fMLF-induced FPR1 phosphorylation was observed. However, PAF induced the release of 40% of PMN lactate dehydrogenase, implying significant cell lysis. We infer that PAF-induced inhibition of fMLF-dependent FPR1 phosphorylation observed in suspension PMN does not occur in the unlysed adherent PMN. We speculate that although the conditions of the assay may induce PAF-stimulated necrosis, the cell densities on the plates may approach levels observed in inflamed tissues and provide for an explanation of PAF's divergent effects on FPR1 phosphorylation as well as PMN function.

## 1. Introduction

Human polymorphonuclear leukocytes (PMN, neutrophils) are the first line of cellular defense in the blood and tissue [1]. They are the host's protection against microbial pathogens and intrinsically involved in general injury and inflammation, recognizing both pathogen-activating molecular patterns (PAMPS) and damage-activating molecular patterns (DAMPS) [2]. As such, they are essential components of innate immunity [3].

PMN are also equipped to deploy their defensive functions with massive cellular accumulation at sites of inflammation upon activation by diverse stimuli, including bacterial and mitochondrial products, denatured proteins, inflammatory lipids, complement components, and certain cytokines [4]. They actively produce superoxide and secrete preformed polypeptides, lipids, and other agents as well as synthesize new components that protect the host by killing and degrading invading pathogens [5]. One consequence of this protective function is the inappropriate inflammatory injury resulting in neighboring bystander tissue. This collateral and often misdirected damage results in a wide variety of disorders [6, 7]. However, PMN also aid in regulating [8] and dampening such injury by responding to anti-inflammatory lipid mediators, such as lipoxins and resolvins [9], as well as anti-inflammatory cytokines [10, 11].

In their quiescent state, PMN are ferried by blood flow and shear-propelled rolling, to monitor the arrival or production of inflammatory mediators along the vasculature [12, 13]. In this capacity, they act as sentries to alert the host and begin the defense against infection and injury. Ultimate exposure to DAMPS and PAMPS stimulates PMN adherence and migration along and through the endothelial layer [14]. They then continue directed migration to mediator sources through the underlying tissue. A driver of the directed migration is the N-formyl peptides that are released by bacteria [15] or broken mitochondria [16] of injured host cells and thus are PAMPS and DAMPS. In order to negotiate the complex pathways leading to the sources of formyl peptides, human PMN employ G-protein-coupled chemotactic receptors that specifically bind N-formyl peptides and their functional homologs. These receptors are called the N-formyl peptide receptors or FPRs (abbreviated in humans as FPR1, FPR2/ALX, and FPR3) [17]. FPR1 is the highly specific, high-affinity form which guides PMN to the inflammatory sites where they may become terminally activated by aggregated immunoglobulins [18] and other localized stimuli such as autocrine and paracrine platelet-activating factor (PAF) [19] and ultimately where FPR1 is presumed to be turned off by a specific termination or desensitization processes [20, 21].

Recently, we showed that two mAbs generated in our laboratory [22] could be exploited to recognize and report on the phosphorylation state of FPR1 in human PMN after activation by formyl peptides [23, 24]. In heterologous systems, FPR1 phosphorylation has been shown to dampen the response to formyl peptides, reducing the effectiveness of fMLF-occupied FPR1 interaction with G proteins [25, 26]. This modification of the receptor appears to mediate its surface sequestration [27] by physical association with arrestin [26] and by internalization of the fMLF peptide [28] as an occupied receptor complex [29–30]. However, the regulation of FPR1 by phosphorylation in PMN still remains relatively unstudied.

To date, most experimental examination of receptor processes contributing to termination of the FPR-mediated processes [20, 31] has taken place on purified PMN in buffered suspension. In the current work, we have examined the phosphorylation of FPR1 in cells adherent to the surface of microtiter wells using similar technology that we previously developed for suspension cells. Not only is this experimental model closer to the physiologic state because of the engagement of adherence receptors and other processes involved in adherence but it would also be expected to prevent PMN aggregation observed in cells suspended in liquid media. In this solid phase system, FPR1 might not behave in the same way as in suspension cells and thus merits some exploratory study.

To recognize and report on the phosphorylation state of FPR1 in adherent human PMN after activation by formyl peptides, we exploited two FPR-specific mAbs generated in our laboratory [22, 23]. We found that FPR1, in adherent PMN, was more sensitive to proteolysis than its suspension cell counterpart, producing a 25 kDa fragment, 25K-FPR1, containing both carboxyl-terminal tail epitopes recognized by the FPR-specific mAbs and thus showing sensitivity to fMLF activation of PMNs. This fragment however was not physiologic, lacking sensitivity to metabolic inhibition, to time or temperature, nor to the types of surfaces the PMNs were adherent to. Instead, it appeared to be formed during cell extraction by SDS solubilization at room temperature and its generation in detergent extracts was significantly slowed if the solubilization took place at 4°C in the lithium salt of dodecyl sulfate which does not precipitate out of solution at low temperature (as does sodium dodecyl sulfate). Exploiting this finding, we revisited the inhibition of fMLF-induced FPR1 phosphorylation by the platelet-activating factor, finding there was no inhibition by a range of PAF concentrations administered either before or after fMLF stimulation. Instead, we found a profound stimulation of cell lysis by PAF that may have contributed to its interesting effect on fMLF-induced phosphorylation.

## 2. Materials and Methods

### 2.1. Reagents

Phenylmethylsulfonyl fluoride (PMSF) was purchased from Calbiochem (EMD Millipore). Goat anti-mouse IgG (H + L) DyLight 800-conjugated antibody was from Thermo Scientific (Thermo Fisher Scientific Inc.) and the Odyssey Infrared Imaging Blocking Buffer was from LI-COR Biosciences (Lincoln, NE). Baxter Healthcare 0.9% sodium chloride injection (USP catalog number 2B1323 in Viaflex bags) and bottled sterile water for irrigation (USP catalog number 2F7113) were obtained from Baxter Travenol. RPMI 1640 (without L-glutamine and with or without phenol red) supplemented with 5 mM HEPES (RPMI/H) and Dulbecco's Phosphate-Buffered Saline (DPBS) was obtained from Corning Cell-Gro. Dextran (average molecular weight = 500,000, 20% autoclaved solution) and all other reagents were obtained from Sigma-Aldrich (Saint Louis, MO).

### 2.2. Antibodies

NFPRa and NFPRb, formerly called NFPR1 and NFPR2, are described in publications by Jesaitis and coworkers [21, 22]. These antibodies were epitope mapped to the N-terminal (NFPRa) and C-terminal S/T-rich (NFPRb) regions of the carboxyl-terminal tail, respectively, [23].

### 2.3. Cell Resuspension and Stimulation Buffers

Dulbecco's Phosphate-Buffered Saline (DPBS with calcium and magnesium) and RPMI/H pH 7.4 were used to resuspend and stimulate PMN.

### 2.4. Cell Extraction Buffers

Buffers used to lyse cells and extract FPR were called TS and TL extraction buffers. Their composition was 0.2 M Tris-HCl pH 8 and 2% sodium dodecyl sulfate (TS) or 2% lithium dodecyl sulfate (TL). Both buffers were supplemented with 1 mM PMSF (added just prior to addition to cells), 2 mM MgSO_4_ and benzonase (500 IU/ml) to digest DNA polymers, 1 : 100 diluted Sigma phosphatase inhibitor cocktail (2 and 3) and 1 : 1000 diluted Sigma protease inhibitor cocktail, and 10 *μ*g/ml leupeptin to inhibit proteases and phosphatases that might be released on cell lysis.

### 2.5. Laemmli SDS-PAGE Sample Buffer (2x, Nonreducing)

Final samples were made up by directly adding the samples to an equal volume 0.4% sodium dodecyl sulfate, 0.12 M Tris-HCl pH 6.8, and 20% glycerol with bromophenol blue.

### 2.6. Special FPR Electrotransfer Buffer

Quantitative electrotransfer of FPR1 to PVDF (Immobilon) required 0.19 M Na glycine, 20% methanol, 25 mM Tris base, and 0.02% SDS, pH 8.5.

### 2.7. Isolation of Human PMN

Human PMN were isolated from heparinized venous blood of healthy individuals using endotoxin-free reagents as described by [32] in accordance with protocol JV-K032216 that was approved by the Institutional Review Board for Human Subjects at Montana State University. Briefly, blood was incubated for 30 min at room temperature at a 1 : 1 ratio with 0.9% sodium chloride (Irrigation USP; Baxter Healthcare) containing 3.0% dextran (Sigma D8802-50 ml) to sediment erythrocytes. The leukocyte-containing supernatant was centrifuged at 670*g* for 30 min and resuspended in 35 ml of 0.9% sodium chloride. The cell suspension was underlaid with 10 ml of Ficoll-Paque PLUS (1.077 g/L; GE Biosciences) and centrifuged for 25 min to separate PMN from peripheral blood mononuclear cells (PBMCs). PBMCs were removed by aspiration and erythrocytes were lysed with water (Irrigation USP; Baxter Healthcare) for 15–30 s followed by immediate mixing with equal volume 1.7% sodium chloride. Purified PMNs were centrifuged at 380 ×g, resuspended in RPMI/H, and enumerated by microscopy. In some cases, DPBS with or without 0.1% glucose was used to resuspend cells. Purity of PMN preparations and cell viability were assessed by flow cytometry (FACSCalibur; BD Biosciences).

### 2.8. Production of Cells Adherent to Wells of 96-Well Plates

PMN prepared as described above were resuspended in RPMI at a cell density of up to 5 × 10^6^ per mL. 50 or 100 *μ*L of cell suspension at the appropriate dilution was added to the wells of a 96-well plate usually with a multichannel pipettor following a protocol to synchronize phagocytosis as described by Voyich et al. [32]. Coating of wells with normal human serum, however, overloaded the SDS-PAGE lanes with protein, preventing accurate FPR1 detection, and was thus avoided. The plates were then centrifuged for 8 min, 8°C, at 1500 rpm (500 ×g) with acceleration set at 7 and deceleration set at 5. These adherent cells formed the basis for most assays except where indicated or where special coatings were added prior to addition of cells to the coated wells. These latter methods were used to explore the condition of FPR1 in cells where specific receptor types mediated adherence. For the experiments described, coating was accomplished similar to Nathan [33] by adding 200 *μ*L of 1 mg/mL human IgG and incubating overnight at 4°C, washing 4x with 250 *μ*L DPBS at room temperature, blocking for 1 h at room temperature with 10 mg/ml bovine serum albumin and 1 mg/ml glucose, and washing 8x with DPBS. For some experiments, wells were coated with the lectins concanavalin A (ConA), succinyl-ConA (SConA), and wheat germ agglutinin (WGA) by a one-hour room temperature incubation at 1 mg/mL and washed and blocked as described above. Cells were not centrifuged in lectin- and IgG- coated plates but allowed to settle and adhere for 1 h at room temperature. No significant differences were observed in fMLF-sensitive NFPRb or NFPRa binding or generation of 25K-FPR1 bands, if cells were centrifuged or coated as described. After coating and addition of cells on to these types of plates, the adherent cells were treated as described for uncoated wells below.

Lactate dehydrogenase (LDH) release to cell supernatants was used to monitor cell lysis in microplate assays. After the indicated incubations (or in parallel experiments) plates were centrifuged for 8 min at 500 ×g (1400 rpm J6 rotor) as above and 7 *μ*L of RPMI or 1% Triton-X100 (for calculating total cell lysis) was added to each well. The mixture was gently agitated for 5 min followed by a second spin and careful removal of 60 *μ*L of supernatant to a second set of wells. 20 *μ*L of such supernatant or at 5 : 1 dilution with RPMI was added with NADH/sodium pyruvate in sodium phosphate buffer assay mixture described previously [34] and initial rate of NADH oxidation measured spectrophotmetrically in a microplate reader at 380 nm. The normalized ratio of supernatant to total LDH activity was calculated to determine % lysis.

### 2.9. Assay for FPR1 Phosphorylation in Adherent PMN

The buffers above the adherent cells were removed using a Rainin pipet-lite 20–200 *μ*L multichannel pipettor. Fifty *μ*L of RPMI was added to the cell-lined wells, followed by 50 *μ*L of a 2 *μ*M solution of fMLF in HEPES-buffered RPMI. The plate was then gently mixed and incubated for 10 min at 37°C. For some experiments, 50 *μ*L of PAF in RPMI of varying dilutions was added and incubated for 5 min at 37°C. In other experiments, the order of PAF and fMLF was reversed but with PAF exposure lasting 10 min. Specific procedures unique to different experiments are provided in the figure legends. After treatments, to examine changes in the state of the cells and FPR1, 100 *μ*L of ice-cold DPBS was added after first removing individual experimental buffers with or without stimulants, inhibitors, or after specific treatments. The DPBS from these “quenched” samples was again carefully removed with a multichannel pipettor. After removal, 100 *μ*L of lithium (TL at 0°C) or sodium (TS at 20°C) dodecyl sulfate extraction buffers was added to iced or room temperature plates, respectively. Plates were then agitated rocking at 20 cycles per min for 10 min at 4°C or room temperature after which 25 *μ*L of freshly made 45 mM DTT (9 mM final) in TS/TL buffer was added and incubated at 60°C for 5 min on a heating block. The plates were then removed from the heating block and cooled for 5 min at room temperature before alkylating for 10 min after addition of 25 *μ*L of freshly made 360 mM NEM (60 mM final) in TS buffer. The samples were then prepared for SDS-PAGE by adding 60 *μ*L of 60°C 2x Laemmli sample buffer containing 30 mg/ml DTT. Thirty to forty *μ*L of these extracts, representing 0.7 to 1.0 × 10^5^ cell equivalents, were either added directly to wells of an SDS-PAGE electrophoresis plate and the remainder frozen in microcentrifuge tubes at −20°C. Alternatively, samples were saved and frozen prior to analysis in a similar fashion. SDS-PAGE and electrotransfer to EMD Millipore Immobilon-FL PVDF Transfer Membranes, immunoblotting, detection, and analysis were described previously by Maaty et al. [23].

## 3. Results and Discussion

### 3.1. Microplate Assay of FPR1 Phosphorylation

To determine if PAF inhibits fMLF-dependent FPR1 phosphorylation without interference from the formation of cell aggregates, we developed a plate assay of FPR1 phosphorylation in PMN adherent to the surface of plastic microtiter plates. We reasoned that if the PMN were adherent, then their immobility would slow the process of aggregation and thus allow better-defined conditions for analysis. In the process of establishing the conditions for assay, we discovered that a polypeptide fragment of 25 kDa was generated that was recognized by two FPR1-specific and epitope-mapped mAbs, NFPRa and NFPRb. These antibodies were produced in our laboratory [22, 35] and used to characterize FPR1 phosphorylation in PMNs in several prior studies. The fragment recognized by NFPRb demonstrated the characteristic NFPRb sensitivity to prior exposure of PMN to fMLF, suggesting it was derived from FPR1 as well as being something we had not observed previously in suspensions of PMN. Since Dahlgren and coworkers have shown that membrane-localized receptor fragments termed pepducins may mediate different interactions with G proteins [36], we felt encouraged to study the origin and relevance of the fragment in more detail.

**Figure 1:**
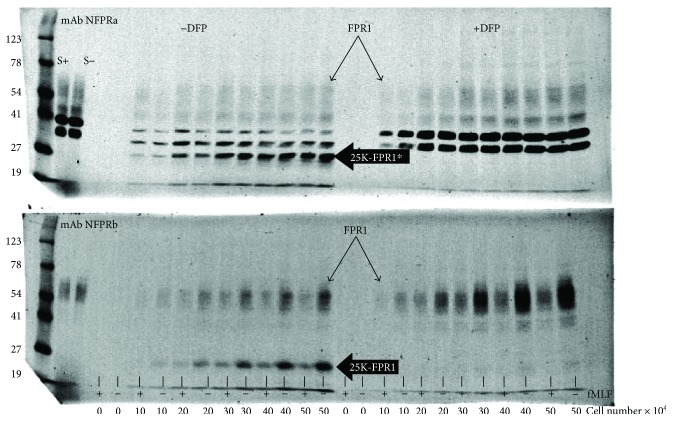
Cell-penetrating serine protease inhibitor diisopropylfluorophosphate blocks FPR1 cleavage in solid phase assay of FPR1 phosphorylation. Twenty-four wells of a 96-well microtiter plate were loaded with 0 to 50 × 10^4^ PMN. Cells had been treated with control (left half) or 3 mM DFP-containing buffer (right half) for 5 min as described in Section 2. The plated PMN were exposed to 1 *μ*M fMLF in RPMI or vehicle control and allowed to incubate for 10 min at 37°C before quenching and washing the wells with 200 *μ*L of ice-cold DPBS followed by extraction as described in Section 2. 40 *μ*L of the final extract (2.7 × 10^4^, 5.3 × 10^4^, 8 × 10^4^, 10.7 × 10^4^, and 13.3 × 10^4^ cell equivalents/lane) was then loaded on two 28-well SDS-poly acrylamide gels, immunoblotted, and developed with NFPRa (upper) or NFPRb (lower) as described in Section 2. Relative molecular mass markers are labeled to the left of the blot. In the two leftmost sample lanes, 1 *μ*M fMLF-exposed (S+) or unexposed (S−) suspension cells prepared as previously described using a comparable cell equivalent load were run as comparative controls. The broad band, of molecular weight ranges 50 to 70 kDa and marked by the two diagonally pointing, single-headed thin arrows, identifies FPR1. The thick, horizontal arrow labeled 25K-FPR1^∗^ upper and 25K-FPR1 shows the position of the putative 25 kDa fragments of FPR1. This species is clearly absent in DFP-treated PMN. The calculated Mr values of the significant bands are FPR1 = 61.4, FPR2 45.3, 39 K 39.7, 35 K 34.7, and 25K 25.6 kDa. Data shown is representative of 2 experiments.

To show that it was possible to measure FPR1 with the numbers of PMN that can adhere to a microtiter plate well, we loaded the wells of a 96-well microtiter plate with up to 5 × 10^5^ PMN and gently centrifuged them directly onto the uncoated plastic. Coatings were at first avoided because preliminary studies indicated that serum coating, for example [32], produced overloaded and uninterpretable gels having anomalous bands and interfering cross-reactivities on immunoblots.  Figure 1 shows immunoblots resulting from solubilization of PMN adherent to an uncoated plate, developed using NFPRa (upper), and NFPRb (lower) as primary mAbs, goat anti-mouse infrared-fluorescing Dylight 800 secondary antibody, and an image from an infrared fluorescence imager. The left and right halves compare PMNs that were untreated or pretreated (resp.) with the cell-penetrating protease inhibitor DFP prior to delivery to the wells of the plate. Each lane corresponds to the solubilized and electrophoretically separated well content having the PMN numbers indicated at the bottom of the blot. Each pair of neighboring lanes corresponds to the solubilized well content after a 5 min exposure of the adherent cells to vehicle (−) or 1 *μ*M fMLF (+).

The *left half of* Figure 1, showing the immunoblots of NFPRa and NFPRb, barely detectable FPR bands can be observed down to 1 × 10^5^ PMN per well. The neighboring pair of control lanes, with no added cells, are blank. At 5 × 10^5^ PMN per well, easily detectable bands are observed and this range of densities down to 1 × 10^5^ PMN/well is approximately linear (not shown). The lower blot, developed using the anti-FPR1 NFPRb primary antibody, shows decreased binding to FPR1 when FPR1 is phosphorylated [22, 23]. This sensitivity is manifested maximally upon PMN exposure to the saturating concentration of 1 *μ*M for 10 min prior to solubilization and is shown in the lanes marked “+” below as compared to paired controls that were exposed to vehicle only and marked as “−”. In these lanes, the 60 kDa FPR1 and 25 kDa bands both show indistinguishable sensitivity to fMLF exposure of PMN (EC_50_ of approximately 20–40 nM), suggesting that the 25K species carries the C-terminal epitope [17, 23, 37] recognized by NFPRb and is therefore a C-terminal proteolytic fragment of FPR1. FPR1 is marked by the diagonal single-headed arrows pointed at the upper boundary of the blot band. The 25 kDa, fMLF-sensitive, putative C-terminal fragment of FPR1 is marked by a horizontal thick single-headed black arrow with the label 25K-FPR1. *Except for the 25 kDa band*, this blot is typical of similar observations made with suspension cells [24]. The control for this experiment is also shown in the left most pair of lanes labeled “S.” Note that no 25 kDa band is observable.

The upper blot shows the bands developed after incubation with NFPRa primary mAb. This antibody recognizes a C-terminal tail region directly adjacent to the predicted 7th transmembrane domain of FPR1 [22, 23]. This region contains no serine/threonine residues and shows no posttranslational modification after exposure of cells to fMLF [23]. In a sense, it is an excellent reference as to how much FPR1 is present in the sample and helps in normalization of sample load in blot analysis. In these blots, one can observe a similar broad band for FPR1 centered at about 60 kDa. It is also marked with the thin diagonal single-headed arrows. The epitope recognized by NFPRa is identically shared by FPR2, which is also observed as a band, less broad and centered at about 45 kDa. It is not a focus of this study. Additionally, there are two very prominent bands at 35 and 39 kDa that are derived from the cytosol [23] and do not show any sensitivity to fMLF. Although they may be of interest to examine in future studies, they also are not a focus for the current study.

A 25 kDa fragment is also observed in the upper left blot depicting the bands recognized by NFPRa. However, as expected, it is not sensitive to fMLF and has the same density of stain irrespective of fMLF treatment of the PMN. The mobilities of the 25 kDa bands, as recognized by NFPRa and NFPRb, are very similar suggesting they represent the same FPR1 fragment. This observation is also supported by examination of their indistinguishable mobilities when identical samples are run on the same gel (not shown). However, we cannot rule out the possibility that part of the density of this band derives from proteolysis of the 39/35 K doublet or some other proteins that binds NFPRa free of the NFPRb epitope but fortuitously matches the mass of C-terminal fragment containing the epitope recognized by both antibodies. To reflect this ambiguity in the NFPRa blots, we call this band 25K-FPR1^∗^. It is also of interest that the 25K-FPR1 and 25K-FPR1^∗^ are not observed in the suspension-prepared PMN shown in the extreme left lanes to the right of the marker lane (labeled “S”). This band was *never* observed to any significant degree for virtually all experiments described in our prior studies on suspension PMN using NFPRa and NFPRb.

### 3.2. 25K-FPR1 Sensitivity to Diisopropylfluorophosphate Treatment of PMN

All our methods of whole PMN extraction, whether by nonionic or ionic detergents, rely on judicious use of protease inhibitors (e.g., PMSF, leupeptin, and commercially available inhibitor cocktails such as from Sigma) to minimize exposure of cellular protein to the high-cellular loads of proteolytic enzymes carried by PMN, estimated to be approximately 5–10% of cellular weight [38]. Nevertheless, certain proteins, such as actin-binding protein, are extremely sensitive to degradation even if the cells are directly solubilized in hot SDS sample buffer in the presence of such inhibitors. To circumvent this problem, Amrein and Stossel [39] utilized a cell-penetrating vital serine protease inhibitor at up to 5 mM, which virtually eliminates postlysis degradation of actin-binding protein in PMN and does not appear to affect their function.

To examine whether DFP blocks the formation of the 25K-FPR1(^∗^) bands, we repeated the experiment described above but after treatment of the PMN with 3 mM DFP. The right half of Figure 1 shows the effect of fMLF and cell load on immunoblots run simultaneously using extracts of PMN treated with DFP and analyzed on the same blot as extracts from untreated PMN shown on the left half of the blot. The right half of the blot shows a more intense banding pattern for the fMLF-sensitive 60 kDa FPR1 band demarking intact receptor recognized by mAb NFPRb, suggesting less proteolysis. Additionally, there is *no observable* 25 kDa band recognized by either antibody. The DFP-dependent elimination of the 25K-FPR1 and 25K-FPR1^∗^, recognized by both NFPRb and NFPRa, respectively, supports the hypothesis that this protein band is a C-terminal fragment of FPR1. Also of interest, but again less relevant to this study, is the enhancement of the heavily stained NFPRa-recognized 39/35 K cytosolic doublet observed in suspension cells mentioned above.

### 3.3. 25K-FPR1 Is Not Physiologic

**Figure 2:**
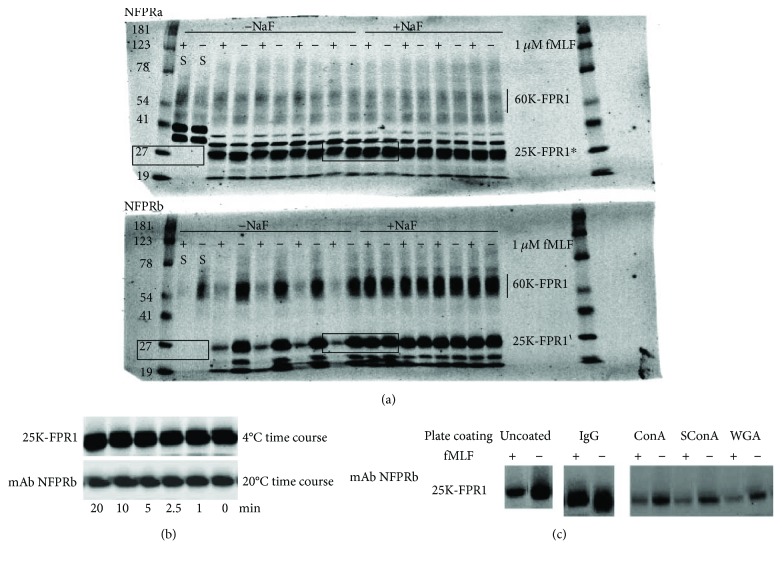
25K-FPR1(^∗^) is insensitive to modulating physiologic conditions. (a) The 25K-FPR1(^∗^) bands persist when adherent PMNs are depleted of ATP. Adherent PMN were incubated with DPBS that contained 40 mM NaF, replacing 40 mM NaCl in the composition of DPBS buffer, prior to treatment with 1 *μ*M fMLF or vehicle for 10 min as described in the legend of Figure 1. The 25K-FPR1 or 25K-FPR1^∗^ bands in line with the Mr 25 kDa are boxed for clarity. The treatment of the cells with or without NaF (+/−) or fMLF (+/−) are so marked in the upper two rows adjacent to their respective labels. The first two lanes, marked “S” adjacent to the 25 K marker (boxed left), show no density in either NaF-treated (+) or untreated (−) PMN indicating that no Mr = 25K bands are being generated by suspension PMN when they are fMLF-treated (+) or untreated (−). The center boxed 4 lanes show the density of the 25 K band in adherent cells for NFPRa (upper) and NFPRb (lower). One of three experiments. (b) The 25K-FPR1 persists unaffected when PMN are allowed to adhere at low temperature. Adherent PMN were not centrifuged but allowed to adhere by settling for 0 to 20 min either at 20°C (lower) or 4°C (upper) before extraction by TS buffer. The 25K-FPR1 was digitally excised from the NFPRb immunoblot scan. The density of the 4°C 25K-FPR1 band appears to be higher than that of the 25K band generated after the 25°C settling assay because they represent different plates incubated at different temperatures. Plate to plate variation is greater than well to well variation. One of two experiments. (c) The 25K-FPR1 persists when PMN are allowed to adhere onto different coatings. Wells of 96-well microtiter plates were incubated with 100 *μ*L of 1 mg/ml autologous human IgG, ConA, succinyl ConA (SConA), wheat germ agglutinin (WGA), and protein-free DPBS. The 25K-FPR1 band was digitally excised from the NFPRb immunoblot scans and is displayed showing the density of the 25K-FPR1 from adherent cells stimulated (+) and unstimulated (−) with 1 *μ*M fMLF. Scanned blots showing different coatings were derived from different experiments on different days and so are not correlated in density with one another. One of four experiments.

If adherent PMN processed FPR1 differently than suspension cells, producing pepducin-like functional fragments of FPR1 [36], it would be of significant biological interest for understanding PMN chemoattractant responses. Thus, with the aim of testing physiologic conditions that might be expected to perturb to receptor processing, we examined the effect of metabolic energy state, time and temperature of adherence, and adherence substrate on the generation of the 25K-FPR1 fragment. Overall, the treatments had little effect on the cell loads in each well indicating that cells were not preferentially retained or lost after treatment. One minor exception occurred with lectin coatings (see below). Figures 2(a), 2(b), and 2(c) show that altering these conditions has very little effect, if any, on the generation of the 25K-FPR1. In Figure 2(a), we depleted PMN of ATP by treatment with NaF for 20 min as previously described [40]. When such ATP-depleted adherent PMN were examined (+NaF), it is clear that unlike untreated cells (−NaF), NFPRb binding to the 25K–FPR1 fragment does not change after fMLF stimulation (see the center boxed region to compare +NaF and −NaF side by side), mirroring what occurs with FPR1 both in adherent (see 60 K-FPR1 above boxed areas) and suspension [24] cells. Since low ATP prevents phosphorylation of FPR1 and thus preserves the unphosphorylated FPR1 C-terminus, it remains equally detectable by mAb NFPRb. This result indicates that the PMN were functioning as previously described, failing to phosphorylate FPR1 without metabolic ATP available. NFPRa binding, not being sensitive to FPR1 phosphorylation, was unaffected by NaF treatment (see the boxed area in the upper blot), suggesting that the 25K-FPR and 25K-FPR1^∗^ bands were still present irrespective of ATP depletion and their generation was not an energy-dependent process.

PMN adherence to substrates is a complex process that might be expected to influence chemoattractant receptor processing and recognition at the cell surface. Therefore, we also examined time and temperature of PMN adherence to the plastic surface to see whether temperature could be seen as facilitating production of 25K-FPR1 in unstimulated cells. Neither of these parameters had an effect on the level of detection of 25K-FPR1 by NFPRa and NFPRb, suggesting the 25K-FPR1 is not produced by a physiologic process of adherent cells.  Figure 2(b) shows that at either 4°C or 20°C allowing cells to adhere for up to 20 minutes had little effect on the amount of 25K-FPR1 detectable by this antibody. There appears to be slightly more 25K-FPR1 generated by a 4°C incubation than that at 20°C, but these two experiments were performed on separate days and would not be expected to match in density as they were developed manually and probably experienced density perturbing variation. Nevertheless, the clear-cut appearance of the 25K-FPR1 and its insensitivity to the time allowed for adherence to occur at the two temperatures suggest that adherence physiology does not appear to play a role in the production of the proteolytic fragment. This observation is clearly different than the electrophoretic profile of FPR1 previously observed in extracts of suspension cells where no 25K-FPR1 is observed under a wide variety of conditions [24].

Receptor-mediated adherence via Fc receptor occupancy by surface immunoglobulin, or by lectin-mediated carbohydrate binding, might involve accelerated membrane and receptor recycling and endocytosis by PMN that potentially could result in modifications of endocytosed membrane components such as FPR1. To examine FPR1 modulation in cells where membrane receptors mediate adherence, stimulate membrane remodeling and cell activation of PMN were also allowed to adhere to microtiter plate wells coated by the lectins ConA, its monovalent succinimidyl ester, SConA, and the wheat germ lectin WGA as described in Section 2. These are independent adherence mechanisms, one mediated by PMN Fc receptors, the others by engagement of different glycoproteins and glycolipids, containing terminal sialic acids and N-acetylglucosamines (WGA binders) and mannosyl and glucosyl groups in multivalent (ConA) as well as monovalent (SConA) interactions. All of these conditions had little effect on the generation of 25K-FPR1 or the relative sensitivity of NFPRb binding to the fragment before or after treatment of PMN with fMLF, as is shown in Figure 2(c). We did however observe a small tendency for more 60K-FPR1 and corresponding 25K-FPR1(^∗^) being recovered in the wells coated with lectins after fMLF treatment (not shown). We conclude that since differing mechanisms of adherence, the time or temperature dependence of adherence, or the inhibition of cellular energetics had virtually no or little effect on the formation of 25K-FPR1(^∗^) or the sensitivity of exposure of PMN to fMLF, then 25K-FPR1(^∗^) production is probably not the result of a relevant cell or cell membrane-mediated process.

It is important to reiterate that we have never seen the formation of the 25K-FPR1(^∗^) fragments while carrying out similar experiments on suspension PMN. In such experiments, PMN membranes are first extracted with the nonionic detergent dodecyl maltoside employing an entirely different geometry for diffusion and mixing of the content of extracted cells. Additionally, our observation is not the result of the use of dodecyl maltoside in the extraction of suspension cells, since in control experiments on adherent PMN where TS buffer is supplemented with nonionic detergents, the 25K-FPR1(^∗^) is still observed (not shown). Thus, we conclude that the 25K-FPR1(^∗^) must be an artifact of extraction. It also should be noted that for years 25K polypeptides had been reported as forms of FPR1 or FPR1 fragment candidates [37, 41, 42]. The experiments described in this report suggest that these older studies could have been identifying FPR1 fragments and not intact receptors or fMLF-binding proteins.

### 3.4. Extraction of PMN at 4°C versus at Room Temperature

**Figure 3:**
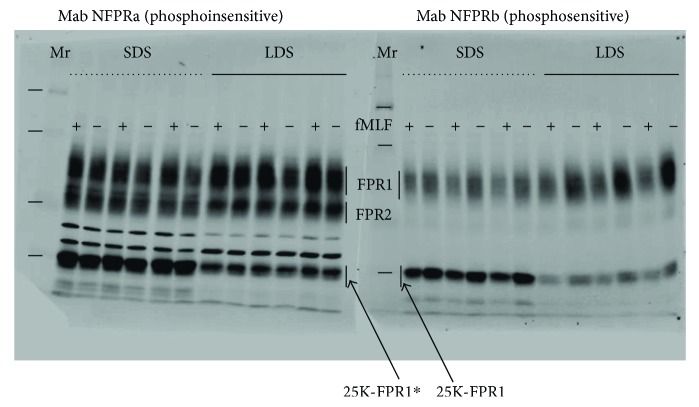
Degree of FPR1 proteolysis depends on conditions of cell solubilization. Six wells on each of two 96-well microtiter plates were loaded with 5 × 10^5^ PMN in 50 *μ*L RPMI buffer and exposed to 1 *μ*M fMLF (+) or RMPI vehicle and equilibrated for 10 min at 37°C as described in Section 2. Subsequently, the supernatants were removed and replaced with 50 *μ*L of ice-cold DPBS. One plate was placed on ice and the other kept at room temperature for 2-3 min followed by careful replacement of the added buffer with ice-cold TL buffer or room temperature TS buffer. The plates were agitated at 4°C and room temperature for 10 min, respectively, and reduced and alkylated normally as described in Section 2. Two identical sets of 12 lanes were loaded with alternating lanes containing (13.3 × 10^4^ ceq/lane) extracts of cells exposed to 1 *μ*M fMLF (+) or control buffer (−) in sets of six. Those that had been extracted at room temperature with TS buffer are denoted with a dotted line and SDS label above. Those extracted at 4°C with TL buffer are denoted with a solid line with LDS label above it. The blotted transfer was cut into two identical halves, incubated with NFPRa (left half) or NFPRb (right half), and developed as described above. The broad band representing FPR1 is identified with a vertical black bar and FPR1 label between the two blots. The position of FPR2 detected by NFPRa is also similarly shown. The 25K-FPR1(^∗^) fragments are identified with diagonal arrows beginning at either 25K-FPR1^∗^ on the NFPRa blot and 25K-FPR1 on the NFPRb blot. The positions of the relative molecular weight markers, Mr, are shown to the left of each blot with horizontal dashes. From top to bottom their values are 125, 78, 41ǂ, and 27 thousand. Each experiment was performed in triplicate or duplicate from two separate PMN donors. (ǂ: omitted for clarity on the NFPRb blot).

Since the proteolytic degradation of FPR1 was nonphysiologic, we attempted to slow the degradation by conducting the extraction of PMN and denaturation of its proteins at reduced temperature.  Figure 3 compares the standard extraction conditions with those at 4°C using lithium dodecyl sulfate as a substitute for its sodium salt in order to prevent precipitation of the detergent at low temperature. The right half of the figure shows a blot from samples run on the same gel developed with phosphosensitive NFPRb and the left half shows identical samples developed with the phosphoinsensitive NFPRa. In each gel triplicate, sets of 5 x 10^5^ PMN per well were exposed to 1 *μ*M fMLF (+) or vehicle (−) and incubated for 10 min before extraction at room temperature with SDS-containing TS buffer shown on the left half of each blot or at 4°C with LDS-containing TL buffer shown on the right half of each blot. In each blot, a more prominent 25K-FPR1(^∗^) C-terminal FPR1 fragment is observed after extraction at room temperature with SDS-containing TS buffer. Although extraction using LDS-containing TL buffer at 4°C did not completely abrogate the formation of the fragment, it greatly reduced it so that the majority of the density was found in the holo-60 kDa FPR1 species. This result suggests that analytical studies using such an assay on PMN should consider using the lower temperature allowed by LDS as the solubilizing agent and thereby avoiding ambiguities introduced by the use of DFP-perturbed PMN physiology. In separate studies using DFP-treated PMN (not shown), we also observed that the 25K-FPR1 had a very similar profile of sensitivity to fMLF as the holo-60 kDa FPR1.

### 3.5. Effect of PAF on fMLF-Induced FPR1 Phosphorylation in Adherent PMN

**Figure 4:**
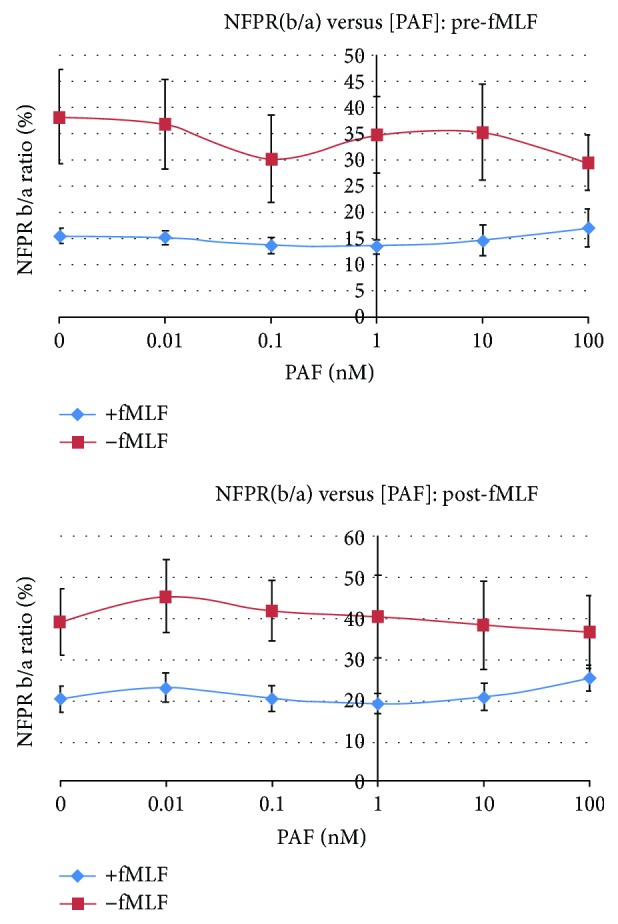
Effect of PAF exposure (pre-fMLF or post-fMLF) on relative FPR1 recognition by NFPR mAbs. Twenty-four wells of a 96-well microtiter plate were loaded with 5.0 × 10^5^ PMN in twelve well sets. The cells were exposed to 50 *μ*L dilutions of PAF ranging from 0 to 100 nM in RPMI and incubated at 37°C for 10 min, followed by addition of 5 *μ*L of 5 *μ*M fMLF (+) or control RPMI buffer and then incubated for an additional 10 min (upper graph labeled [PAF]:pre-fMLF). Alternatively, the order of additions was reversed except that incubation with PAF was for 5 min ([PAF]: post-fMLF). The supernatants were then removed; the cells extracted with TL buffer at 4°C and processed as normally as described in the legend of Figure 3 and Materials and Methods. The 60 K-FPR1 was then quantitated utilizing LI-COR Odyssey Image Studio software. The paired NFPRb/NFPRa integrated intensity ratios were calculated (see Supplement to Figure 4) and plotted as a function of the log of PAF concentration with the zero concentration plotted on the left ordinate. Error bars represent SEM for four measurements using cells from two blood donors. A representative immunoblot and Odyssey quantitation method are shown in Figure 4 Supplement.

Having shown that the 25K-FPR1 fragment results from proteolysis during extraction of FPR1 for immunoblots and that its presence can be minimized by conducting the extraction at 4°C, we attempted to re-examine the effect of PAF on fMLF-induced FPR1 phosphorylation in adherent PMN.  Figure 4 shows the effect of control and 4 logs of PAF concentration on the normalized density of NFPRb binding to FPR1. PAF concentration ranged from zero (control) up to 100 nM PAF and applied either 10 minutes before or 5 minutes after fMLF stimulation of plate adhered PMN. In this experiment, we found that the FPR1 signal varied significantly, as PAF caused variable amounts of sample loss (see below) after removal of supernatants. To mitigate this variability, the normalized NFPRb density was calculated as the *ratio of NFPRb to NFPRa signal for identical samples* and is plotted as a percent. This value is relatively independent of the amount of sample in the wells and provides a measure of the relative density of NFPRb binding compared to NFPRa binding. Normally, after exposure of PMN to 1 *μ*M fMLF, this value decreases 50 to 70% of its maximum control value (−fMLF) because of the phosphorylation-dependent loss (+fMLF) of NFPRb binding [24]. In our last study, we found that in suspension PMN after 100 nM PAF treatment, this value did not decrease with exposure of PMN to 1 *μ*M fMLF [24]. We interpreted this result to mean that FPR1 phosphorylation was inhibited after PAF, even though there was a significant aggregation of the PMN.  Figure 4 shows that with control vehicle treatment, the normalized NFPRb density shown in the upper red curve remains relatively constant for all PAF concentrations. After 1 *μ*M fMLF, the normalized NFPRb density decreased to a value of about 15 from 40, a 62% drop. This post-PAF/post-fMLF treatment remains relatively constant for the full PAF concentration range shown by the blue curve. A similar result was observed when PAF followed fMLF, indicating that phosphorylation was not reversed by PAF. Together, the results suggest that unlike in suspension PMN, PAF-treated PMN are unaffected in FPR1 phosphorylation stimulated by fMLF at all but the highest PAF concentrations. At the highest PAF concentration, 100 nM, it can also be seen from a representative experiment shown in Supplement to Figure 4 and used to derive the numerical data for Figure 4 that lower the levels of NFPRa (i.e., a loss of FPR1 content) accompanies the reduced NFPRb density. This loss probably derives from cell loss as well as additional proteolysis (see below) during processing as well as solubilization. So although it appeared that suspension-type aggregation was avoided because cells were adherent to the plate, PAF caused significant and often visible loss of cells that reduced the receptor load of the wells. Nevertheless, the *normalized* NFPRb density remained relatively unaffected and still showed sensitivity to fMLF.

Delving into these phenomena further, we found that in plate-adherent PMN, there was about a 40% release of lactate dehydrogenase after 100 nM PAF or after 1 *μ*M fMLF followed by 100 nM PAF stimulation (not shown) compared to control levels of 5 to 10%. In further preliminary studies on plated PMN, we also found that although fMLF stimulated a robust continuous and extended production of superoxide as described by Nathan [33], PAF caused it to cease. Moreover, PAF exposure alone did not stimulate superoxide production in the plated cells but caused them to become unable to produce superoxide in response to fMLF stimulation. A process leading to PAF-associated lysis or necrosis would explain these results and begs further study. It also suggests that 60% of the cells remaining intact retained their ability to phosphorylate FPR1 after fMLF exposure but did not produce superoxide. We speculate that the differences between our results on PAF-treated, plate-adherent neutrophils, PAF-stimulated suspension cells, and the PAF-induced reactivation of fMLF-dependent superoxide production by PMN reported by Forsman et al. [43] stem from the conditions of assay and their influence on PMN function. Forsman and coworkers carried out their measurement at 10^5^ cells/mL; our suspension PMN studies were carried out at a 50–100-fold higher densities, while for most of the experiments reported in the current study, the number of cells per well was 4 × 10^5^. The approximate center to center distances for the PMN for these 3 configurations differ by as much as 60-fold (10 *μ*m for plates, 120–150 *μ*m for our suspension experiments, and 600 *μ*m for PAF-induced reactivation). Signal weakness precludes study at significantly lower plate PMN densities; however, the densities of cells employed here matched the much more closely packed densities of PMN observed in injured, infected, and inflamed tissues. Further study of the dynamics of PMN FPR1 at high-cell densities may help in understanding how PMN function as they populate sites of infection, injury, and inflammation.

## 4. Conclusion

The hallmark of inflammatory injury is a massive influx of PMN to sites of infection, injury, and inflammation at close to the nearest neighbor distances. FPR1 is a GPCR that recognizes the PAMPS and DAMPS released by infecting and injured cells and that drives PMN to such sites and high densities. The current study employs cell densities that match those found in inflamed tissues much more closely than studies of suspension PMN and shows that even though PMN begin the process of lysis induced by high PAF, their ability to phosphorylate FPR1 remains mostly intact. It also shows that the study of molecular processing of FPR1 in such PMN by immunological methods is possible, if one takes exceptional care to inhibit proteolysis. Lastly, this study also describes the methodology that might be employed to get a closer molecular look at stimulus-response coupling in cells designed to overwhelm tissues and microorganisms with the arrival of their massive numbers outfitted with damage-inducing but host-defensive responses.

## Supplementary Materials

Supplementary Materials Clarification of Figure 4 calculation.
