# Supplementary material for: Formyl Met-Leu-Phe-Stimulated FPR1 Phosphorylation in Plate-Adherent Human Neutrophils: Enhanced Proteolysis but Lack of Inhibition by Platelet-Activating Factor
Source: J Immunol Res. 2018 Jan 24;2018:3178970. doi: 10.1155/2018/3178970 (PMC5896419; doi:10.1155/2018/3178970)
Supplement: Supplementary Materials — Clarification of Figure 4 calculation. [file 3178970.f1.docx]

**Supplementary material**

**for**

Formyl Met-Leu-Phe-stimulated FPR1 phosphorylation in plate-adherent human neutrophils: Enhanced proteolysis but lack of inhibition by platelet activating factor

Algirdas J. Jesaitis^*^, Jeannie Gripentrog, Jovanka M. Voyich

Department of Microbiology and Immunology

Montana State University

Bozeman MT, 59715

**Figure 4 Supplement**

**
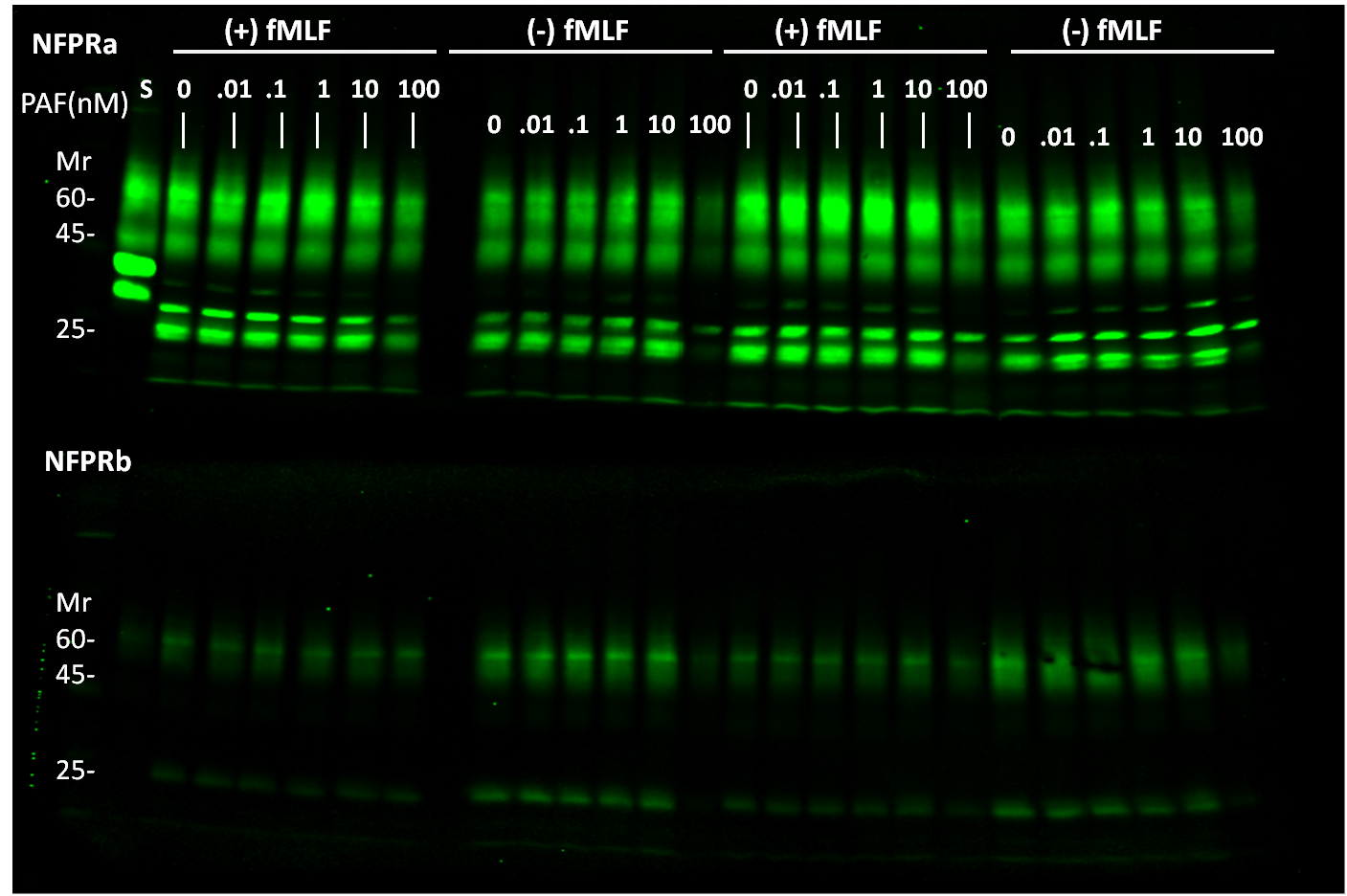
**

**

**

**Supplement to Figure 4 Legend --**Clarification of Figure 4 calculation.

The Figure 4 supplement shows representative NFPRa and NFPRb immunoblots used to calculated data points in Figure 4. It is one of the two donor experiments carried in duplicate, in this case for fMLF for 10 min preceding PAF at the indicated concentrations.

**Fig 4 supplement A** shows two immunoblots of identical samples developed as described in Materials and Methods using NFPRa (upper) and NFPRb (lower). Wells all contained 4 x 10^5^ PMN treated as described in the legend to Figure 4, in this case with exposure to fMLF preceding PAF exposure. Each lane is labeled with a concentration of PAF that the well was exposed to and whether it had been also exposed to fMLF(+) or fMLF vehicle (-), in this case before PAF.

**Figure 4 supplement B** shows the quantitation system that Licor Odyssey software uses to calculate the densities of bands in lanes of gels or immunoblots. Because PAF induces some cell losses (see text) it was important to normalize cell loads. For the experiments described in Figure 4, the normalized NFPRb density was calculated by dividing the NFPRb integrated intensity (lower box) by the NFPRa integrated intensity (upper box in the corresponding lane). These normalized NFPRb values were used to assess the phosphorylation state of FPR1 in the fMLF (+) samples to those in the fMLF (-) samples. These values were averaged for each PAF concentration and plotted as shown in Figure 4 for 4 replicates experiments using 2 donors. The calculation from this experiment (one donor) is shown in the table below:

| PAF | fMLF | NFPRa | NFPRb | NFPR(b/a) |
| --- | --- | --- | --- | --- |
| nM | 1µM |  |  |  |
| 0 | + | 366337 | 94215 | 0.257 |
| 0.01 | + | 337393 | 93743 | 0.278 |
| 0.1 | + | 374169 | 99984 | 0.267 |
| 1 | + | 393920 | 95760 | 0.243 |
| 10 | + | 348791 | 98021 | 0.281 |
| 100 | + | 232615 | 91181 | 0.392 |
|  |  |  |  |  |
| 0 | - | 240738 | 150568 | 0.625 |
| 0.01 | - | 244307 | 155789 | 0.638 |
| 0.1 | - | 263977 | 158653 | 0.601 |
| 1 | - | 286833 | 150509 | 0.525 |
| 10 | - | 308308 | 164119 | 0.532 |
| 100 | - | 141020 | 78751 | 0.558 |
|  |  |  |  |  |
| 0 | + | 392994 | 102107 | 0.260 |
| 0.01 | + | 384216 | 106159 | 0.276 |
| 0.1 | + | 441095 | 107460 | 0.244 |
| 1 | + | 442096 | 111543 | 0.252 |
| 10 | + | 379096 | 115081 | 0.304 |
| 100 | + | 264038 | 92982 | 0.352 |
|  |  |  |  |  |
| 0 | - | 334466 | 190074 | 0.568 |
| 0.01 | - | 320127 | 158304 | 0.495 |
| 0.1 | - | 358454 | 126356 | 0.353 |
| 1 | - | 304850 | 169233 | 0.555 |
| 10 | - | 281400 | 167706 | 0.596 |
| 100 | - | 148633 | 84801 | 0.571 |
